# Knowledge, use (misuse) and perceptions of over-the-counter analgesics in sub-Saharan Africa: a scoping review

**DOI:** 10.1080/16549716.2021.1955476

**Published:** 2021-08-23

**Authors:** Rachel Kawuma, Rujeko Samanthia Chimukuche, Suzanna C Francis, Janet Seeley, Helen A Weiss

**Affiliations:** a Social Aspects of Health Programme, MRC/UVRI and LSHTM Uganda Research Unit, Entebbe, Uganda; b Social Science and Research Ethics Department, Africa Health Research Institute, KwaZulu-Natal, South Africa; c MRC International Statistics and Epidemiology Group, Department of Infectious Disease Epidemiology, London School of Hygiene and Tropical Medicine, London, UK; d Department of Global Health and Development, London School of Hygiene and Tropical Medicine, London, UK

**Keywords:** Over the counter, analgesics, self-care, self-medication, Sub-Saharan Africa, non-prescription drugs

## Abstract

**Background:**

Over-the-counter (OTC) analgesics are safe for pain-management when used as recommended. Misuse can increase the risk of hypertension and gastrointestinal problems.

**Objective:**

To conduct a scoping review of the uses and misuses of OTC analgesics in sub-Saharan Africa, to inform strategies for correct use.

**Method:**

Following guidelines for conducting a scoping review, we systematically searched Pubmed, ResearchGate and Google Scholar databases for published articles on OTC analgesic drug use in sub-Saharan Africa, without restrictions on publication year or language. Search terms were ‘analgesics’, ‘non-prescription drugs’, ‘use or dependence or patterns or misuse or abuse’ and ‘sub-Saharan Africa’. Articles focusing on prescription drugs were excluded.

**Results:**

Of 1381 articles identified, 35 papers from 13 countries were eligible for inclusion. Most were quantitative cross-sectional studies, two were mixed-methods studies, and one used qualitative methods only. About half (*n* = 17) the studies recorded prevalence of OTC drug use above 70%, including non-analgesics. Headache and fever were the most common ailments for which OTC drugs were taken. Primary sources of OTC drugs were pharmacy and drug shops, and family, friends and relatives as well as leftover drugs from previous treatment. The main reasons for OTC drug use were challenges in health service access, perception of illness as minor, and knowledge gained from treating a previous illness. Information regarding self-medication came from family, friends and neighbours, pharmacies and reading leaflets either distributed in the community or at institutions of learning. OTC drug use tended to be more commonly reported among females, those with an education lower than secondary level, and participants aged ≥50 years.

**Conclusion:**

Self-medicating with OTC drugs including analgesics is prevalent in sub-Saharan Africa. However, literature on reasons for this, and misuse, is limited. Research is needed to educate providers and the public on safe use of OTC drugs.

## Background

Analgesics obtained over-the-counter (OTC) such as paracetamol, ibuprofen and aspirin are widely used to manage pain including fever, headache, musculoskeletal pain and menstrual cramps [1,2]. They are safe if taken as recommended, but misuse has been associated with conditions such as hypertension and gastrointestinal tract infections [3,4]. Many people use OTC drugs as their first line of treatment, without formal prescription, because they are affordable and accessible from small drug stores, street vendors and friends and family [5].

Obtaining medication without the authority or prescription of a physician enables people to access treatment quickly and reduces the burden on the health care system [6–8]. However, if not used as recommended, OTC drugs can result in wasted medical resources and harms related to side effects, drug toxicity, drug-drug interactions, and drug-disease contraindications [9–12]. More positively, OTC drug use is a component of self-care, reflecting the ability of individuals, families and communities to promote, maintain health, prevent disease and to cope with illness with or without the support of a health care provider [13]. Once equipped with the correct information, people can make decisions to manage their health [7,14,15].

Globally, research on OTC drug use has been conducted among specific groups such as students [8,16,17], pregnant women [18] and children [19,20], as well as the general population [9,21]. This research has covered use of range of OTC drugs in high income countries [2,22], but little research has been conducted about the knowledge, use (and misuse) and perceptions of OTC drugs including analgesics, particularly paracetamol and ibuprofen in low-income settings, including in sub-Saharan Africa. In a systematic review conducted in 2017, it was noted that OTC drug use including analgesics was prevalent in Ethiopia [7]. A study in South Africa showed that OTC analgesics were used for intravaginal insertion to increase sexual pleasure and other unintended purposes leading to substance abuse dependency [23], while a study on menstrual health in secondary school girls in Uganda, that some expressed concern about the effects of analgesics on fertility [24].

To map the prevalence and different forms of OTC analgesic use and misuse in sub-Saharan Africa and perceptions of such use, we undertook a scoping review. Scoping reviews use a structured process to map the coverage of a given topic in the literature and to identify knowledge gaps [25–27]. The findings of this scoping review will identify research gaps on OTC analgesic drug use, and inform future research on strategies to improve the use of OTC analgesics in the region.

## Methods

### Search strategy

Following scoping review guidelines [26], potentially eligible articles were identified through PubMed, Research gate and Google scholar. The search was conducted in January 2020, with terms as follows:

1) (‘analgesically’[All Fields]) OR ‘analgesics’[Pharmacological Action]) OR ‘analgesics’[MeSH Terms]) OR ‘analgesics’[All Fields]) OR ‘analgesic’[All Fields] OR ‘Non prescription drugs’ [All fields] OR Paracetamol OR Panadol OR aspirin OR ibuprofen OR analgesic*)

2) (use or Dependence or Patterns or misuse or abuse)

3) ((((((((((‘africa south of the sahara’[MeSH Terms] OR ((‘Africa’[All Fields] AND ‘south’[All Fields]) AND ‘sahara’[All Fields])) OR ‘africa south of the sahara’[All Fields]) OR ((‘sub’[All Fields] AND ‘saharan’[All Fields]) AND ‘Africa’[All Fields])) OR ‘sub-Saharan africa’[All Fields] OR (Cameroon OR ‘Central African Republic’ OR Chad OR Congo OR ‘Democratic Republic of the Congo’ OR “Å‘Equatorial Guinea’ OR Gabon OR ‘Sao Tome and Principe’ OR Burundi OR Djibouti OR Eritrea OR Ethiopia OR Kenya OR Rwanda OR Somalia OR ‘South Sudan’ OR Sudan OR Tanzania OR Uganda OR Angola OR Botswana OR Lesotho OR Malawi OR Mozambique OR Namibia OR ‘South Africa’ OR Zambia OR Zimbabwe OR Benin OR ‘Burkina Faso’ OR ‘Cabo Verde’ OR ‘Cote d’Ivoire’ OR ‘Ivory Coast’ OR Gambia OR Ghana OR Guinea OR Guinea-Bissau OR Liberia OR Mali OR Mauritania OR Niger OR Nigeria OR Senegal OR ‘Sierra Leone’ OR Togo)))))).

There was no restriction on the study design, language or year of publication. Further articles were identified from reference lists of eligible papers.

### Data abstraction

We included publications reported data on the use (or misuse) of OTC analgesics in sub-Saharan Africa. We excluded publications that focused on prescribed drugs, animal-based studies and studies conducted outside of sub-Saharan Africa. For each potentially eligible article identified, two authors (RK and RSC) abstracted the following information to identify eligible papers: study title, author, year of publication, country, study design, sample size, category of users, prevalence of self-medication with OTC analgesics and other drugs, common illnesses treated, common OTC drugs used and sources from which they were obtained, sources of information and the reasons for medication.

## Results

**Figure 1.**
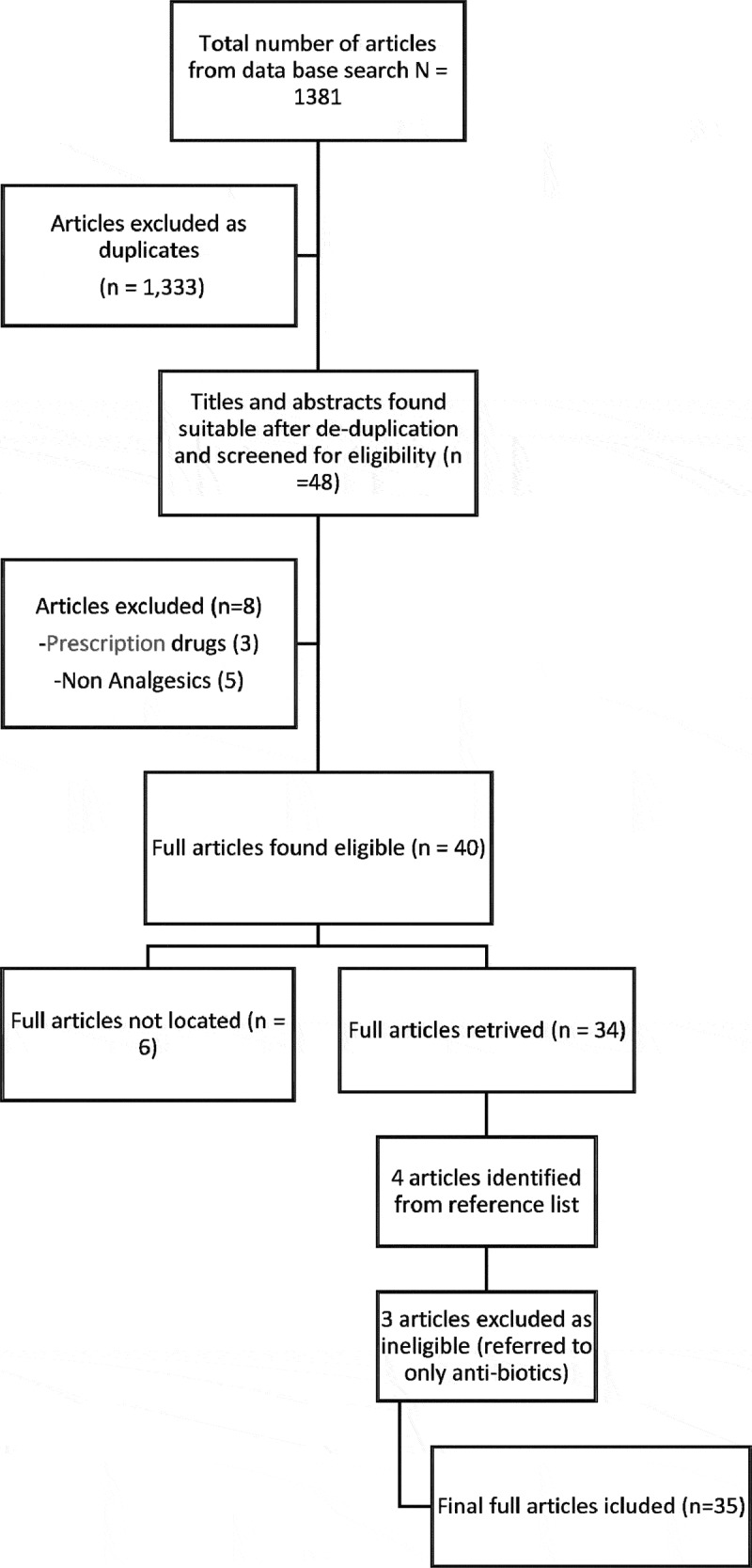
Flow diagram of study selection

A total of 1381 studies were identified from the database search. After removing duplicates, 48 studies were deemed potentially eligible, and their abstracts were reviewed using the inclusion and exclusion criteria above. Forty abstracts were found eligible for inclusion. Three were excluded because they involved prescription drugs and five did not specifically refer to analgesic use. Of these, 34 full-text papers were found. We were unable to obtain the papers for the remaining six articles through libraries or by trying to contact the authors. An additional four potentially eligible papers were identified from reference lists of eligible papers. Of these, one met the eligibility criteria, yielding a total of 35 papers included in the analysis (Figure 1).

### Characteristics of studies included

The 35 articles were published between 1989 and 2019, from 13 countries in sub-Saharan Africa. Study populations ranged in size from 57 to 9063 participants (total number of participants = 27,257). Thirteen studies were conducted in Nigeria, with others in Ethiopia (*n* = 9), Ghana (*n* = 2), Tanzania (*n* = 2) and one each from South Africa, Gambia, Mozambique, Eritrea, Zimbabwe, Democratic Republic of Congo, Cote d’Ivoire, Cameroon, and Kenya. Populations included secondary and university students (*n* = 6 studies), pregnant women (*n* = 6), among children and adolescents (*n* = 4), general populations (*n* = 13) and hospital-based studies including health care professionals (*n* = 2), and adult outpatients and caregivers (*n* = 4). Most studies used quantitative methods (*n* = 32), with two using mixed methods and one qualitative methods only (in-depth interviews and focus group discussions) (Table 1).

**Table 1.** Study characteristics arranged by country and design

First Author and Year	Country	Design	Sample size and population	Methodology
Afolabi 2004 [28]	Nigeria	Cross-sectional study	1943 sick children	Record of patient’s diagnosis
Yusuff 2011 [29]	Nigeria	Cross-sectional study	1650 pregnant women	Structured questionnaire
Adelekan 1989 [30]	Nigeria	Cross-sectional study	1000 secondary school students	Self-administered questionnaire
Abasiubong 2012 [31]	Nigeria	Cross-sectional study	518 pregnant women, aged 18–40 years	Structured questionnaire
Enato 2011 [32]	Nigeria	Cross-sectional study	497 heads of household	Questionnaire
Nwankwo 2010 [33]	Nigeria	Cross-sectional study	495 post-menarcheal school girls aged 10–19 years	Semi-structured questionnaire
Bello 2011 [34]	Nigeria	Cross-sectional study	410 women attending antenatal	Self-administered questionnaire
Oshodi 2010 [35]	Nigeria	Cross-sectional study	402 secondary school students	Self-administered questionnaire
Esan 2018 [36]	Nigeria	Cross-sectional study	384 undergraduate university students	Self-administered questionnaire
Lawan 2013 [14]	Nigeria	Cross-sectional study	380 adults	Structured questionnaire
Babatunde 2016 [15]	Nigeria	Cross-sectional study	291 healthcare workers	Self-administered questionnaire
Obu 2012 [37]	Nigeria	Cross-sectional study	231 caregivers to children aged six weeks to 16 years	Self-administered questionnaire
Omolase 2007 [38]	Nigeria	Cross-sectional study	200 hospital outpatients	Structured questionnaire
Amberbir 2011 [39]	Ethiopia	Population-based prospective birth cohort	1065 pregnant women	Face to face interviews in a longitudinal study
Amberbir 2014 [40]	Ethiopia	Population-based prospective birth cohort	1006 newborn children	Face to face interviews with mothers in a longitudinal study
Birru 2016 [41]	Ethiopia	Cross-sectional study	720 students	Self-administered questionnaire
Beyene 2018 [42]	Ethiopia	Cross-sectional mixed methods study	617 pregnant women 9 key informants	Structured questionnaire and IDI guide
Shafie 2018 [9]	Ethiopia	Cross-sectional study	604 heads of households	Structured questionnaire
Amaha 2019 [12]	Ethiopia	Cross-sectional study	577 adults	Structured questionnaire
Duncan 2006 [43]	Ethiopia	Cross-sectional mixed methods study	204 members of the general population for questionnaires and 8 FGDs (number of participants unspecified)	FGDs and questionnaires
Eticha 2014 [6]	Ethiopia	Cross-sectional study	270 community members	Structured questionnaire
Sado 2017 [44]	Ethiopia	Cross-sectional study	154 health professionals	Self-administered questionnaire
Marwa 2018 [45]	Tanzania	Cross-sectional study	372 pregnant women	Self-administered questionnaire
Chipwaza 2014 [46]	Tanzania	Cross-sectional study	93 community members and 14 healthcare workers	FGDs and IDIs
Mensah 2019 [47]	Ghana	Cross-sectional study	361 community members	Self-administered questionnaire
Badzi 2017 [48]	Ghana	Cross-sectional study	206 construction workers	Structured interviews
Myers 2003 [49]	South Africa	Retrospective study	9063 patients from specialist substance abuse treatment centre	Assessment of patients’ prescription forms
Clarke 2003 [50]	Gambia	Cross-sectional study	917 women	Structured questionnaire
Lucas 2007 [51]	Mozambique	Cross-sectional study	797 university students	Self-administered questionnaire
Tesfamariam 2019 [52]	Eritrea	Cross-sectional study	609 adults	Structured questionnaire
Kasilo 1991 [53]	Zimbabwe	Cross-sectional study	498 household members	Questionnaire
Ndol 2013 [54]	Democratic Republic of Congo (DRC)	Cross-sectional study	391 hospital patients	Questionnaire
Angbo-Effi 2011 [55]	Cote d’Ivoire	Cross-sectional study	300 adult household members	Questionnaire
Penda 2018 [56]	Cameroon	Cross-sectional study	295 hospitalised patients aged 0–18 years	Semi-structured questionnaire
Geissler 2000 [57]	Kenya	Cross-sectional study	57 schoolchildren aged 11–17 years	Face to face interviews

### Prevalence, common ailments, drugs used and their sources

Of the 35 studies, 28 recorded prevalence of OTC drug use with both analgesic and non-analgesic drugs. In this paper we focus on the OTC analgesic use. Overall, 17 studies recorded a recall period ranging from one week to 12 months. The remaining studies did not indicate a recall period. Seventeen studies (60.7%) had a prevalence of OTC analgesic use above 70%. Specifically, prevalence among pregnant women was between 26–78%, students 55–82%, general community members 38–97% and hospital patients 45–89%. Twenty-one studies described common ailments treated with OTC analgesic drugs, namely headache (*n* = 15), fever (*n* = 13), cough and cold (*n* = 10), diarrhoea (*n* = 6) and general body/joint pains (*n* = 5). These were similar by type of study population.

Paracetamol was the main OTC analgesic drug used, others were aspirin and ibuprofen. Paracetamol is generally considered to be a non-toxic drug without known side effects and a drug people can easily obtain. It was used to treat common illnesses such as headaches, fever, cough and gastrointestinal infections. Many of the papers included OTC drug use with other classes of drug, such as antibiotics, antimalarial and anthelminthic drugs.

The main sources of OTC drugs were pharmacies and drug shops in the community (*n* = 12), family, friends or other neighbours (*n* = 9), drug hawkers (*n* = 4), left over medication from previous treatments (*n* = 4) and patent medical stores (*n* = 3) (Table 2).

**Table 2.** Prevalence of OTC drug use, drugs used, sources of OTC drugs and common ailments treated

Author and Year	Prevalence of OTC drug use	Recall period	Common drugs used	Sources of drugs	Illnesses or conditions treated
Yusuff 2011 [29]	64%	90 days	Paracetamol (31%) Haematinics and vitamins (23%) Promethazine (8%) Piroxican (8%) Diazepam (8) Amoxicillin (5%) Dipyrone (5%) Chloramphenicol (4%) Ampicillin (4%) Panadol extra (3%) Procold (16%)	Patent medicine stores (55%) Pharmacies (31%) Drug hawkers (15%)	Body pains/fever (30%) Joint pains (15%) Cough (10%) General weaknesses (9%) Indigestion (9%) Headache (8%) Insomnia (8%) Nausea (7%) Heartburn (3%) Inflammation (2%)
Adelekan 1989 [30]	58%	NR	Salicylate analgesics (58%) Alcohol (18%) Stimulants (34%) Antibiotics (18%) Other (10%)	NR	NR
Abasiubong 2012 [31]	72%	Current pregnancy	Analgesics 157 (30%) Antibiotics 138 (27%) Mixed herbs & other drugs 47 (9%) Sedatives 15 (3%) Alcohol (3%) Kolanut (1%)	NR	NR
Bello 2011 [34]	78%	NR	Acetaminophen (48%) Antimalarial (6%) Anthelminthic (69%) Calcium supplements (1%)	NR	NR
Esan 2018 [36]	82%	1 month	Paracetamol (75%) Ibuprofen (12%) Aspirin 5%) Other (8%)	Doctor (3%) School clinic (3%) Hostel (1%) Home (3%) Other (1%)	Headache (46%) Stomach ache (7%) Body pain (15%) Muscle pain (4%) Dysmenorrhoea (12%) Fever (3%) Cough/cold (3%) Arthritis pain (1%) Other (4%)
Lawan 2013 [14]	79%	6 months	Antimalarials (42%) Analgesics (41%) Antibiotics (29%) Cough mixtures (13%) Other (6%)	Patent medical stores (63%) Markets (20%) Drug hawkers (12%) Family/friends (6%)	NR
Babatunde 2016 [15]	52%	12 months	Analgesics (38%) Antibiotics (19%) Antimalarials (13%) Other (29%)	NR	Body pains (15%) Catarrh (15%) Headaches (14%) Sore throat (12%) Diarrhoea (11%) Fever (9%) Toothache (6%)
Omolase 2007 [38]	85%	NR	Antimalarials (16%) Antibiotics (3%) Antimalarials and analgesics (22%) Antimalarials, analgesics and antibiotics (15%) Antibiotics and analgesics (10%) Antimalarials and antibiotics (6%) Anti-hypertensive (1%) Hypoglycaemic (1%)	NR	NR
Amberbir 2011 [39]	29%	1 month	Paracetamol (100%)	NR	Asthma (2%) Hay fever (4%) Eczema (1%)
Amberbir 2014 [40]	60% in the first 3 years of life; 35% for current exposure at 5 years old	12 months	Paracetamol (100%)	NR	Fever (31%); Headache(24%) Common cold (7%) Malaria 4%) Wheezing illness (4%) Coughing illness (1%) Allergy (1%)
Birru 2016 [41]	73%	12 months	Paracetamol (64%) Diclofenac (25%) Ibuprofen (8%) Migraine-specific agents (4%)	Previous treatment (33%) Pharmacy (45%) Physician (7%) Non-drug substances (5%) Other (26%)	Headache (100%)
Beyene 2018 [42]	27%	Current pregnancy	Paracetamol (49%) Amoxicilin (23%) Ibuprofen (5%) Panadol (6%) Diclofenac (5%) Aspirin (3%) Other (10%)	Pharmacy/drug stores (77%) Leftovers (19%) Family/friends or neighbours (10%) Health facilities (1%)	NR
Shafie 2018 [9]	76%	2 months	Antacids (5%) Anthelminthic (6%) Antibacterial (15%) Cough syrup (0%) Traditional herbs (16%) Combination drugs (8%) Not specified (1%)	Pharmacies/drug stores (83%) Neighbours (7%) Previous treatment (7%) Other (3%)	Headache (26%) Abdominal pain (13%) Cough (12%) Diarrhoea (9%) Toothache (8%) Combination (6%) Other (26%)
Amaha 2019 [12]	38%	1 month	Antibiotics (41%) Analgesics (27%) Anthelminthic (14%) Antacids (12%)	Pharmacy/drug stores (64%) Friends/relatives and neighbours (24%) Other (11%)	Gastro-intestinal disease (29%) Urinary tract infection (12%) Eye and skin infection (31%) Fever (20%) Other (8%)
Eticha 2014 [6]	88%	NR	Analgesics (21%) Gastro-intestinal drugs (18% Respiratory drugs (15%) Oral rehydration solution (14%) Vitamins (11%) Antimicrobial (8%)	NR	Headache or fever (21%) Gastro-intestinal (17%) Respiratory infections (16%) Eye disease (14%) Skin disease (13%) Dysmenorrhea (11%) Sexually transmitted diseases (5%) Other (3%)
Sado 2017 [44]	68%	2 months	Analgesics (36%) Antibiotics (24%) Oral contraceptives (17%) Antacid (13%) Oral hypoglycemic agents (7%) Other (4%)	NR	NR
Marwa 2018 [45]	46%	Current pregnancy	Antimalarial (25%) Antibiotics (10%) Antiemetics (34%) Analgesics (19%) Anti-asthma (2%) Other (11%)	NR	Malaria (33%) Urinary tract infection (9%) Morning sickness (25%) Heartburn (2%) Headache (20%) Other (14%)
Chipwaza 2014 [46]	NR	NR	Antimalarial* Antipyretic* Antimicrobial*	Community Pharmacies Family and friends and neighbours Previous treatment	Fever Diarrhoea Cough Loss of appetite and ‘flu’ Abdominal pain Wound and headache
Mensah 2019 [47]	NR	3 Months	Antibiotics (32%) Analgesics (21%) Antidiabetic (20%) Antimalarial (10%) Antacids (11%) Antihypertensive (5%)	Licenced chemists (32%) Traditional practitioners (26%) Family/friends (21%) Community pharmacy (20%)	NR
Badzi 2017 [48]	97%	NR	Paracetamol (37%) Aspirin* Ibuprofen (10%) Combined analgesics (52%)	Pharmacy stores (58%) Chemical stores (86%) Drug peddlers (4%) Friends (12%)	Muscle and joint pain* Headaches* Stress* Fatigue*
Myers 2003 [49]	45%	NR	Analgesics*	NR	NR
Clarke 2003 [50]	NR	NR	Paracetamol (28%) Chloroquine (8%)	Pharmacy Market traders Private drug outlets Village shops	Fever (94%) Vomiting (79%) Headache (33%) Diarrhoea (27%)
Lucas 2007 [51]	56%	1 month	Analgesics (38%) Anti-infectives (15%) Antimalarials (6%) Vitamins (8%) Oral contraceptives (5%)	NR	NR
Tesfamariam 2019 [52]	94%	1 month	Analgesics (34%) Antipyretics (16%) Cough and cold preparations (14%) Antacid (10%) Antidiarrheal (10%) Vitamins (8%) Anti-allergy (5%) Anti-inflammatory (2%) Other (1%)	NR	Non-chronic disease (72%) Chronic diseases (25%) Central nervous system disorders (1%) Other (1%)
Kasilo 1991 [53]	95%	NR	Analgesics (50%) Respiratory drugs (21%) Dermatological (9%) Gastrointestinal (8%) Antimalarial (7%) Eye ointments (3%) Other (1%)	Chemist/ pharmacist (56%) Shop/ Supermarket (23%) Hospital/clinic (19%) Other (2%)	Sore throat/ cough/colds (16%) Pain (9%) Stomach pain (6%) Skin infection (5%) Fever (5%) Sore eyes (2%) Cramps/ muscle pain (2%) Other (28%)
Ndol 2013 [54]	60%	NR	Analgesics/Antipyretics (44%) NSAIDS (26%) Antimalarials (21%) Antibiotics (12%)	NR	NR
Angbo-Effi 2011 [55]	72%	NR	Analgesics (75%) Antimalarials (72%) Antibiotics (48%)	NR	NR
Penda 2018 [56]	74%		Antipyretics (75%) Antimalarials (72%) Antibiotics (10%) Anthelminthic (6%) Other (23%)	Family pharmacy box (64%) Pharmacy (22%) Street vendors (14%)	Fever (83%) Vomiting (20%) Diarrhoea (20%) Cough (13%) Headache (9%)
Geissler 2000 [57]	NR	1 week	Antimalarials (14%) Antibiotics (4%) Paracetamol (66%) Aspirin (24%)	Family/friends/ Neighbours* Shop keepers*	Headache (30%) Abdominals (12%) Colds (13%) Other (28%)
Afolabi 2004 [28]	89%	NR	Analgesics/Antipyretics (63%) Antimalarial (23%) Antibiotics (21%) Haematinics/vitamins (55%) Cough syrup (12%) Local herbs (9%) Antihistamines (5%) Antidiarrheals (4%) Anthelminthic (2%) No drug (11%)	NR	Fever (61%) Cough (44%) Diarrhoea (21%) Loss of appetite (17%) Vomiting (17%) Catarrh (16%) Other (52%)
Enato 2011 [32]	45%	2 Weeks	Antidiarrheal (2%) Antimalarial (90%) Antibiotic (3%) Analgesics (3%) Antipyretic (<1%) Anticonvulsant (<1%) Antihistamine (<1%) Surgical operation (<1%)	General hospital (9%) Private hospital (9%) Primary health care (14%) Traditional herbal medicine (1%) Maternity home care (16%) Treated home (1%) Patent medicine store (6%) Self-treatment (45%)	Alimentary (10%) Cardiovascular (1%) Dermatological (1%) Nervous system (58%) Infections (10%) Musculoskeletal (5%) Respiratory (15%) Sensory organs (1%)
Nwankwo 2010 [33]	43%	NR	Paracetamol* Aspirin/Piroxican (80%) Antibiotics (19%) Other (1%)	NR	Dysmenorrhea (25%) Premenstrual disorder (19%) Cycle less than 21 days (12%) Cycle greater than 45 days (5%) Scanty menstruation (5%) Prolonged bleeding (4%)
Obu 2012 [37]	76%	NR	Paracetamol (100%)	Patent medical stores/pharmacy (4%) Self (45%)	Fever (63%) Cough (7%) Abdominal pain (4%) Other (14%) Non-response (12%)
Duncan 2006 [43]	NR	1 month	Aspirin (44%) Paracetamol*	NR	Headache* Fever*
Oshodi 2010 [35]	NR	NR	Paracetamol* Aspirin* Antimalarials*	NR	NR

NR = Variable not recorded; *Percentages not reported

### Reasons, sources of information and socio-demographic characteristics for OTC drug use

In 19 studies, reasons for use of OTC drugs were explored. These related to challenges in the healthcare system such as long distances to the health facilities, long queues and limited health service staff. Drugs from pharmacies, hawkers or nearby shops without a prescription provided more ready access. In addition, the perception of illnesses as being minor and treatable with OTC drugs was mentioned in 13 of the studies. Other reasons were financial constraints (n=6) involving paying consultation bills but also the knowledge gained from previous treatments (n=5).

Thirteen studies recorded the main sources of information for OTC drug use and participants reported family members, friends and neighbours as the main source of information. Other sources were the pharmacies where they went to buy drugs, and drug information leaflets. The media, including the internet, were also an information source, including through advertisements.

Table 3 shows socio-demographic characteristics associated with use of OTC drugs analysed in 16 studies. Five studies analysed the relationship between age and use of OTC drugs and majority showed that participants aged ≥40 years used OTC drugs more than the younger participants. However, one study found that younger participants used OTC drugs less than older participants (70% among those aged 14–40 years vs 50% for those aged ≥50 years) [54]. Six studies reported that females use OTC drugs more often than males while one study conducted among children found out that boys used more drugs than girls [57][48]. There was also a correlation between education and OTC drug use, with six studies finding that respondents with low levels of education (mostly below secondary school level) tended to use OTC drugs more than those with higher levels of education [12, 14, 15, 34, 45, 52]. In contrast, two studies found that those with lower level of education were less likely to use OTC drugs than those with higher levels of education [31, 47] (Table 3).

**Table 3.** Reasons, sources of information and socio-demographic characteristics for OTC drug use

Author and Year	Reasons for OTC use	Sources of knowledge or information OTC use	Socio-demographic characteristics of OTC use
Yusuff 2011 [29]	Accessibility/uncontrolled availability (40%) Long distance to public health facility (30%) Financial difficulty (19%) Perceived poor service delivery at facility (12%)	Mothers-in-law and relatives (41%) Patent medicine vendors (20%) Pharmacist (13%) Nurse (10%) Neighbour (8%) Traditional healer (7%)	NR
Adelekan 1989 [30]	Cost saving (22%) Influence from others (33%) Mild severity of illness (18%) Bureaucracy (23%)	NR	Gender: More OTC use among females than males*
Abasiubong 2012 [31]	NR	NR	Education: More OTC use among participants with more education (25% vs 7%)
Bello 2011 [34]	NR	NR	Education: More OTC drug use among participants with less education* Age: More OTC drug use among older than younger participants*
Esan 2018 [36]	Unfriendly attitude of health care workers (28%) Lack of time to go to school clinic (27%) School clinic is too far from hostel (15%) Don’t trust quality of drugs (15%)	NR	Gender: More OTC use among females than males (88% vs 71%)
Lawan 2013 [14]	Long queues (38%) Doctors not available (25%) Services too expensive (18%) Not necessary (to consult doctors for prescription) (19%)	Drug vendors* Family/friends* Self* Other*	Age: More OTC use among older participants (aged ≥40 years) than among younger participants* Education: More OTC use among participants with below secondary education*
Babatunde 2016 [15]	Financial problems (11%) Mild sickness (11%) Lack of time (13%) Knowledge of diagnosis (6%) Convenience (2%) Non-availability of doctor (3%)	NR	Age: More OTC use among older (49 years above) participants than the younger ones* Education: More OTC use among participants with less education*
Omolase 2007 [38]	Complaint is minor (55%) Financial constraint (22%) Services not readily available (9%) Certainty of efficacy of self-medication 10%) Lack of escort (3%) Ignorance (1%)	NR	NR
Amberbir 2014 [40]	Readily available (77%) Affordable (92%)	NR	NR
Birru 2016 [41]	NR	NR	Gender: More OTC drug use among females than males*
Beyene 2018 [42]	Easy access (71%) Disease not serious (55%) Saves time (27%) Cheaper (18%) Previous experience (21%) Poor health service provision (1%) Long waiting time (20%)	NR	NR
Shafie 2018 [9]	Minor illness (47%) Prior knowledge of the drug (23%) Emergency case (11%) Time constraint (6%) Long queues (2%)	Health professional (45%) Previous treatment (21%) Friends (16%) Self (13%) Reading books/internet (4%)	Age: More OTC drug use for older participants (≥55 years) than younger participants* Income: More OTC drug use for low earners than higher earners* Gender: More OTC use for females than males (54% vs 22%)
Amaha 2019 [12]	Minor illness (48%) Quick relief (79%) Long queues (59%) Attitude of health workers (8%) Health facilities expensive (13%) Long distance (28%)	Friends, relatives or neighbours (59%) Labels, leaflets or promotional materials (8%) TV advertisement (9%) Internet (7%) Past experience (37%)	Education: More OTC use among participants with less education* Income: More OTC use for high earners than low earners*
Eticha 2014 [6]	Disease was not serious (22%) Prior experience of the illness and/or the drug (21%) Less expensive or time-consuming (20%) Emergency care (17%) Prevention of known or unknown illness (17%) Symptoms of illnesses (17%)	Pharmacists (23%) Health workers (21%) Friends, neighbours or relatives (19%) Reading drug-related information (13%) Traditional healers (13%)	NR
Sado 2017 [44]	Familiarity with drug (24%) Mildness of illness (14%) Privacy (17%) Less cost/financial constraint (33%) Lack of time (7%)	NR	NR
Marwa 2018 [45]	NR	NR	Education: More OTC use among pregnant women with secondary education and below* Occupation: More OTC use among unemployed than among employed*
Chipwaza 2014 [46]	Shortage of drugs at health facilities* Long waiting times at health facilities* Long distance to health facility* Unable to pay for health care costs* Freedom to choose drug of choice*	Parents/guardians* Pharmacy/Drug shop/vendors* Health workers*	NR
Mensah 2019 [47]	Cost saving (2%) Influence from others (friends/family) (33%) Mild severity of illness (18%) Bureaucracy of health system (23%) Other (4%)	NR	Education: More OTC use among participants with more education*
Badzi 2017 [48]	Prescribed (7%) Induce sleep (27%) Relieve aches and pains (66%)	Advertisements (73%) Friends (64%) Prescriptions (7%) Other (2%)	NR
Myers 2003 [49]	NR	NR	Gender: More OTC use among females than males)*
Clarke 2003 [50]	Hospital services too expensive* Long distance to health facility*	NR	NR
Lucas 2007 [51]	NR	NR	Gender: More OTC use among females than males (65% vs 42%)
Tesfamariam 2019 [51]	Ease of accessibility (34%) Saving time (24%) Perception of being safe and tolerable (15%) Saving money (6%) Treating minor ailments (4%) Getting quick relief (4%)	Pharmacists (35%) Medical doctors (27%) Friends/family (21%) Internet/mobile applications (3%)	Education: More OTC use among participants with less education*
Kasilo 1991 [53]	Long queues at hospitals* Long waiting time at hospitals*	NR	NR
Ndol 2013 [54]	NR	NR	Age: More OTC use among younger participants than older patients (71% for those aged <30 years; 59% for those 31–50; and 50% for those ≥ 50) Marital status: More OTC use among those who lived as couples self-medicated than singles (66% vs 55%)
Angbo-Effi 2011 [55]	Accessibility (13%) Cheaper than other options (69%) Lack of finances (16%)	Influence from others (54%)	NR
Penda 2018 [56]	Mildness of illness (56%) Disease severity (27%) Persistent disease (52%) Financial problem (7%)	Drug information leaflets (75%) Pharmaceutical advice (65%)	NR
Geissler 2000 [57]	NR	NR	Gender: More OTC drug use among males than females (34% vs 9%)
Afolabi 2004 [28]	Perception of illness (96%)	NR	NR
Enato 2011 [32]	NR	Family members, neighbours and friends (62%) Clinic (1%)	NR
Obu 2012 [37]	NR	Self (45%) Doctor (20%) Nurse (7%) Patent medical dealer/pharmacist (4%)	NR
Oshodi 2010 [35]	NR	NR	Gender: More OTC use among females than males*

NR = Variable not recorded; *Percentages not reported

Misuse of OTC drugs was explored in few studies. In two studies, participants reported that higher than recommended doses were taken [31,52]. A third study highlighted perceptions about drug misuse that can result in health hazards such as drug resistance [14].

## Discussion

This is the first scoping review to assess the knowledge, use (misuse) and perceptions of OTC analgesics in sub-Saharan Africa. OTC analgesics use is widespread in sub-Saharan Africa to treat common ailments such as fever and headaches. OTC analgesics were obtained mostly from pharmacies and drug shops. Challenges with the formal health care system were cited as the main reason for using OTC drugs, along with perceptions of ailments as minor and not requiring treatment in a formal system.

The 35 studies included in this review provided evidence of a high prevalence of OTC drug use including analgesics in comparison to studies conducted in general populations outside Africa [58–60] which suggest a lower prevalence of self-medication of less than 20%, except among university students [3,4,17,61]. However, comparison is difficult because of differences in the demographic profile of study populations, recall periods, methodologies used and a lack of uniformity in defining ‘self-medication’ may have contributed to observed prevalence rates, a factor which Cooper [22] identifies as a challenge in a review of the global use (misuse) of OTC drugs (including analgesics). In addition, it is important to recognise that lax regulatory practices governing drug shops and pharmacies, as well as access to affordable health services, also influence OTC drug use and misuse [62,63].

In our review, OTC drugs including analgesics were used by all populations, including children and adolescents, students and pregnant women as has been noted in studies elsewhere [5,8,19,60]. The main reasons for use were to treat febrile illnesses at home and to avoid seeking medical attention unless symptom persisted. OTC drugs including analgesics were easily accessible from several sources including close friends and relatives. This easy accessibility, together with challenges in the formal health care services such as the long distances and absence of dedicated personnel explained the high prevalence of OTC drug use in the region [64, 65].

Perceptions of risks involved in the misuse of OTC drugs was explored in three studies [14, 31, 52]. These found that people mainly misused drugs by taking more than the recommended dosage. Misuse of OTC drugs is understudied in sub-Saharan Africa despite risks that may be associated with harms including addiction, drug resistance and masking underlying symptoms of an illness [2,11,66]. A global systematic review [22], which included only two studies from Africa [49,67], noted that OTC drugs were misused. More studies need to be conducted to understand the misuse of OTC analgesics to ensure that they are used responsibly, as advocated for by the World Health Organisation [13].

Quantitative cross-sectional surveys were the most common study design used to investigate OTC drug use; however, qualitative methods could offer in-depth insights into misuse of OTC drugs. Further, there were few studies from East or Southern Africa.  Evidence from these regions could help understand patterns of use and misuse of OTC analgesics across the sub-Saharan African region to inform policies to promote responsible self-care.

The differential use of OTC drugs by socio-demographic characteristics is useful for formulation of interventions and policies. For instance, females tended to report self-medication more than males and this is partly attributed to pains related with dysmenorrhea [33,66,68], but this may also reflect reporting bias, with males less likely to disclose analgesic use [69]. People with lower levels of education also tended to use OTC drugs, including analgesics, more than those with higher levels of education [12,14,15,34,45,52]. However, there were some exceptions, including prevalent OTC use by university medical students, as seen elsewhere [3,16,35,44]. The ease of access to drugs was given as the main reason why health workers, including medical students, took drugs without prescriptions raising concerns over misuse [11]. Therefore, regardless of education level, health education may need to be emphasised to support people to learn appropriate use of OTC drugs while eliminating their misuse.

A limitation of our review was that studies reported prevalence of both OTC analgesic drugs and non-analgesics combined, making it difficult to elicit analgesic use specifically.

## Conclusion

OTC drugs, including analgesics, are commonly used in sub-Saharan Africa by men and women (including pregnant women), children and people from different geographical locations (both rural and urban) to manage febrile illnesses which some people perceived to be minor and treatable. However, while they can be easily accessed, their use and perceptions are not well-understood, especially in terms of misuse because this was understudied. Therefore, limited knowledge regarding misuse of OTC drugs was noted in this scoping review. Further research is needed to understand the specific use and misuse of analgesics in sub-Saharan Africa to inform better practice, and the reasons for increased reporting by females and those with less education. We recommend that studies (including studies using qualitative methods) focusing on OTC analgesics use specifically (rather than OTC drug use generally) be conducted to understand reasons for high prevalence and whether such use is appropriate. These can inform development of national guidelines, essential medicine lists, educational programmes and other effective mechanisms to promote rational medicine use by consumers.

## Paper context

There is limited research on the use and misuse of OTC analgesics in low-income settings. We conducted the first scoping review on OTC analgesic use in sub-Saharan Africa, and found high prevalence of self-medication. This is likely to be due to ease of access and challenges accessing formal health care. Further research, including studies using qualitative methods, are needed to help understand patterns of use and misuse of OTC analgesics in the region, to inform policies to promote responsible use.
